# The effects of sociability on exploratory tendency and innovation repertoires in wild Sumatran and Bornean orangutans

**DOI:** 10.1038/s41598-017-15640-x

**Published:** 2017-11-13

**Authors:** Caroline Schuppli, Sofia Forss, Ellen Meulman, Suci Utami Atmoko, Maria van Noordwijk, Carel van Schaik

**Affiliations:** 1 Department of Anthropology, University of Zürich, Winterthurerstrasse 190, 8057 Zürich, Switzerland; 2 Fakultas Biologi, Universitas Nasional, Jl. Sawo Manila, RT.14/RW.3, Ps. Minggu, DKI Jakarta, Indonesia

## Abstract

It has been hypothesized that opportunities for social learning affect the size and complexity of the adult skill set of birds and mammals, their learning ability, and thus ultimately also their innovation frequency. To test these predictions we compared rates of social learning, rates of independent exploration (independent learning) and innovation repertoires between individuals of a highly sociable population of *Pongo abelii* at Suaq Balimbing and a less sociable population of *Pongo pygmaeus wurmbii* at Tuanan. Suaq immatures showed significantly higher rates of peering, even after controlling for differences in association time and diet complexity, implying that they make disproportionally greater use of their increased opportunities for social learning. As predicted, we found that immatures and adults at Suaq also showed significantly higher rates of exploratory behaviour. The difference between the individuals of the two popuations remained when controlling for association time, suggesting persistent developmental effects, intrinsic differences, or both. Accordingly, Suaq animals had a larger set of learned skills and a higher mean dietary complexity. Our findings show that population level sociability, individual rates of exploration and population-wide repertoires of innovations are positively linked, as predicted.

## Introduction

Cultural effects on cognitive development in humans have been documented for a long time and have reached the status of common knowledge: social inputs during childhood have a strong effect on the development of the cognitive skill set^1^. Studies on institutionalized children have shown that children raised with limited social inputs show deficits in a variety of domains, including language, social-emotional development and intelligence^2,3^. These effects are also evident in structural and functional changes in the brain^4,5^. Recent studies have shown that later cognitive performance is also affected by more subtle differences in the frequency and quality of social inputs during critical periods of early childhood^6^: increased social interactions during day care^7^, a higher degree and consistency of parents’ responsiveness during early childhood^8,9^ and early education and care programs^10^ were all found to have significant positive effects on cognitive development and later cognitive abilities in various domains including problem solving, inhibitory control, memory and language.

Experiments on a variety of nonhuman species suggest that similar processes might also be at work in other animals, suggesting some degree of evolutionary continuity. On the one hand, extreme social deprivation during development has been shown to lead to smaller adult skill sets and reduced learning abilities in several mammal taxa^11–14^. At the other extreme, enculturation (treating infants as if they were human babies) is known to lead to a more rapid development of a broader set of skills as well as increased learning ability in primates, in particular apes^15,16^. Thus, as in humans, social inputs during development affect individuals’ subsequent cognitive performance as well as the number of skills they have accumulated by adulthood. However, there has been little investigation so far of the underlying mechanisms or the degree of evolutionary continuity in the more fine-grained effects of the quality of social inputs on cognitive development. Research on nonhuman primates can help to fill this gap.

It is generally accepted that an important part of the explanation of these effects is that social learning is more efficient than independent learning. Experiments have shown that social learning leads to faster skill acquisition^17–20^. Social learning leads subjects to focus on the relevant information and allows for skill acquisition without time-consuming and potentially dangerous innovation. Furthermore, skills can be passed on to the next generation through social learning, making skill repertoires heritable. These repertoires are a clear target for natural selection as opposed to the learning ability per se, which will only sometimes result in innovation and thus actual fitness benefits in the absence of social inputs. Consequently, the net benefit per unit brain tissue is higher in lineages with social learning and it should be easier for selection to favour the evolution of increased learning ability in species that rely on social learning compared to species that solely rely on independent learning^21^.

The interspecific correlation of independent- and social-learning abilities^22^ suggests that they may rely on overlapping sets of basic cognitive mechanisms^23^. Furthermore, in most forms of social learning, only the trigger is social, whereas the association itself is most likely dependent on forms of independent learning^21,23^. Also, a growing body of evidence suggests that even observational forms of social learning in nature mostly entail a phase of independent practice of the observed behaviour^24–27^. The two learning mechanisms also interact: experiences and abilities gained through social learning are likely to be transferred to new situations and thus increase overall cognitive performance^21,28^.

The close links between the two learning mechanisms suggest that selection on social learning will automatically also favour the evolution of the ability to learn independently. This phenomenon is consistent with the cultural intelligence hypothesis, which predicts effects of opportunities for social learning on cognitive abilities, in both humans^29,30^ and social animals more generally^21,31^. First, on the developmental (proximate) level the cultural intelligence hypothesis predicts that individuals with more opportunities for social learning during development will acquire larger sets of learned skills as adults. Through experience effects, and based on the cognitive overlap between the two learning mechanisms, increased opportunities for social learning during development will also lead to an enhanced independent learning ability. Second, on an evolutionary (ultimate) level, the cultural intelligence hypothesis states that species with consistently more opportunities for social learning will eventually evolve an increased motivation to engage in social learning and the innate ability (and thus brain size) to do so, which goes hand in hand with higher exploratory tendencies and thus results in an increased likelihood of innovation. Thus, populations containing more opportunities for social learning should exhibit overall larger and more complex repertoires of learned skills compared to populations with fewer opportunities.

The aim of this study was to investigate the effects of varying opportunities for social learning on exploration by looking at direct effects of associations on exploration rates in wild orang-utans. These effects will give insight in how population differences in sociability may further cause differences in exploratory tendeny and skill repertoires between populations. Orang-utans represent the ideal natural experiment for this because their degree of sociability varies significantly between populations, with Sumatran orang-utans, *Pongo abelii*, generally spending more time in association than Bornean orang-utans, *Pongo pygmaeus* ^32,33^. Whereas in the Bornean populations studied to date mother-offspring pairs spend on average around 15% of their time in association with at least one other individual, in the known Sumatran populations they do so for 25–45% of their time^34^. The increased association frequency of Sumatran orang-utans provides immatures and adults with more opportunities to learn from a greater number of conspecifics. Orang-utans rely on a broad range of learned skills in the ecological context and it takes immatures more than ten years to learn the full range of these skills^35^. Surprisingly, despite showing broad repertoires of learned skills, wild orang-utans are far more novelty averse and show lower rates of exploration than those in human care^36,37^. Accordingly, immatures learn their skills socially, through peering (attentive close range watching of a conspecifics activity) at knowledgeable individuals such as their mothers and others^24,27^. In orang-utans, social learning and independent exploration are tightly linked: peering elicits an immediate increase in exploration of the same object or food item as the peered-at individual is manipulating^24,27^. Thus, sociability and opportunities for social learning might also have direct effects on exploration rates.

Besides these direct effects of social inputs on exploration, developmental effects and intrinsic differences may also contribute to differences in exploratory tendency. In terms of organisational developmental effects, being exposed to increased levels of sociability over the course of development may have lasting effects on an individual’s overall exploratory tendency, independent of its current state of association. Over evolutionary time, those developmental effects may have become genetically founded and resulted in intrinsic differences in individuals’ exploratory tendencies independent of developmental inputs. Therefore, it is important to separate the direct effects of association on exploration rates from those of developmentally induced organisational or innate differences in exploratory tendency between the two species.

We tested the following predictions of the effects of increased sociability on innovation through a comparison of two orang-utan populations with major differences in sociability: the highly sociable Suaq Balimbing population (*P. abelii*) and the less sociable Tuanan population (*P. pygmaeus wurmbii*). First, assuming that social learning is more efficient than independent learning, we predict that individuals at Suaq make use of their increased opportunities and show higher rates of indicators of social learning (i.e. peering; cf.^27^). Second, we predict that increased opportunities for social learning will result in higher rates of exploratory behaviour at Suaq. Since immature vertebrates are generally more exploratory than adults^38–42^, these differences should be most pronounced in immatures. To rule out that these differences are not simply a result of generally increased activity levels in the more sociable population, we will use the frequency of other types of object manipulation behaviour as a control (for definitions see methods and Table 1). Third, in order to identify the mechanisms involved, we will separately examine the direct and the developmental effects of associations on exploratory tendency. If there are direct effects of association on exploratory tendency, we predict that individuals will show increased rates of exploration when in association. If increased levels of sociability have lasting developmental (or even evolutionary) effects, we predict that individuals in the more sociable population will show increased exploration rates, even as adults and when on their own. Since direct, developmental and intrinsic effects are not mutually exclusive, we might find both of these predictions confirmed. Finally, on the population level, we predict that increased exploratory tendencies will result in a higher likelihood of innovations and thus larger repertoires of learned skills as well as higher skill complexity.

**Table 1:** Definitions and classifications of the focal behaviours.

Behaviour	Definition
Exploratory object manipulation	Prolonged, non-repetitive, usually destructive manipulation of, or feeding attempts on, objects (such as fruits, sticks, leaves, other plant or material, animal products, etc.), excluding actual ingestion. The visual and tactile focus of individual is on the object, accompanied by an intent facial expression.
Playful object manipulation	Manipulation of an object without an apparent goal. These manipulations are often repetitive but brief and variable. The visual and tactile focus are desynchronized or only transiently synchronized.
Peering	Direct and sustained (at least 5 seconds) intense watching of the action of another individual, at a close enough range to be able to observe the actions in detail (usually less than 2 m).

## Methods

### Study sites

Data were collected from 2007–2014 at Suaq Balimbing (3°42′N, 97°26′E, Aceh Selatan, Indonesia) on individuals of a population of wild Sumatran orang-utans and from 2012–2015 at Tuanan (2°09′S, 114°26′E, Kalimantan Tengah, Indonesia) on individuals of a population of wild Bornean orang-utans. Both study sites consist of peat swamp forest and are highly comparable in terms of their ecology. With 7.4 individuals per km^2^, Suaq shows the highest-recorded Sumatran orang-utan density, whereas Tuanan’s density, despite being the highest for Borneo, is significantly lower with 3.8 individuals per km^2^ ^43^. These differences in density are thought to be directly related to differences in food availability^44^.

### Data sets

We followed 21 different immature individuals (13 at Suaq, aged 0.5–14.5 years and 8 at Tuanan, aged 0.5–11.3 years) as well as 11 (5 and 6) of their mothers as adult references. All focal individuals were well habituated and known to the researchers since the mothers have been followed for many years. For the immatures, data collected within 5 months were averaged to create a single age-specific individual mean. We refer to these as an “observation period”. This resulted in 18–19 age/individual observation periods of immatures for Suaq and 9–11 age/individual observation periods for Tuanan, depending on the observed behaviour (see above). The complete data sets used for the analyses can be found in the appendix (Table S1a and b).

Immature individuals were classified as (i) dependent immatures who are between birth and weaning (infants). Among the weaned immatures (juveniles), we distinguished between (ii) semi-independent immatures, who are weaned but still in permanent association with their mothers and (iii) independent immatures, who are no longer in permanent association with their mothers. Weaning age at Suaq is around 7–9 years and at Tuanan around 6–7.5 years.

### Data collection

Behavioural data were collected following an established protocol for orang-utan data collection (http://www.aim.uzh.ch), using focal animal sampling, including instantaneous scan sampling at two-minute intervals. This instantaneous data includes the activity of the focal individual, in case of feeding or object manipulation the involved item and species, the distance to all association partners, in case of social interactions the interaction partner, the height of the focal individual in the tree as well as several other parameters. Additionally, all object manipulation behaviour and indicators of social learning were described in detail, including context, exact description of the type of manipulation and the manipulated object or interaction partner (Table 1). We followed the definition of Hutt for the distinction between explorative and playful object manipulation^40,45^. The manipulated object include all food and non food items such as sticks, leaves, fruits, branches etc. The vast majority of the manipulated objects were ubiquitous at both sites.

Data were collected by CS at both sites as well as by SF, EM and several well-trained observers. Inter-observer reliability was assessed through simultaneous follows by multiple observers on the same focal animal without verbal exchange about the activity of the focal animal. Only data of observers that reached an index of concordance with CS of more than 85% on the frequency of the specific focus behaviour were included in the analysis. This led to slight variations in sample size, depending on the observed behaviour.

Associations were defined as two or more individuals being within 50 meters to each other. At every two-minute interval during scan sampling, the distance between the focal individual and all association partners was assessed.

### Data Analyses

All analyses and plots were done using the R programming language^46^. Data were analysed with linear mixed-effects models with a Gaussian probability distribution, using lmer as implemented in the package lme4^47^. The model fits were examined visually for deviations from the data, satisfaction of assumptions, and presence of influential observations^48^. To stabilize the variance and meet the model assumptions, in some models the response variable was log transformed (models 2a, 2c, 3, 4a and 5) or square root transformed (models 2b and 4b). To account for the fact that data were collected on the same immature individuals over multiple periods, at different stages of their development, we included the individual as a random factor. The significance of the predictor variables (“site” for models 1a + b, 2 a-c, 3, 4a + b, 5 and 6; as well as “complexity” and “frequency” for model 3, “sex” for model 4 and “context” for model 6) was assessed by excluding the specific factor from the model and comparing the full models to the reduced model using the likelihood ratio test (using the “anova” function in R). The factors “age” or “age class” were included in the models as control variables to account for the fact that the rates of these behaviours are strongly correlated with age. Depending on the model, the best fit of the age term was either linear or quadratic (age + age^2^), which was assessed using likelihood ratio test.

### Population difference in opportunities for social learning

**Figure 1:**
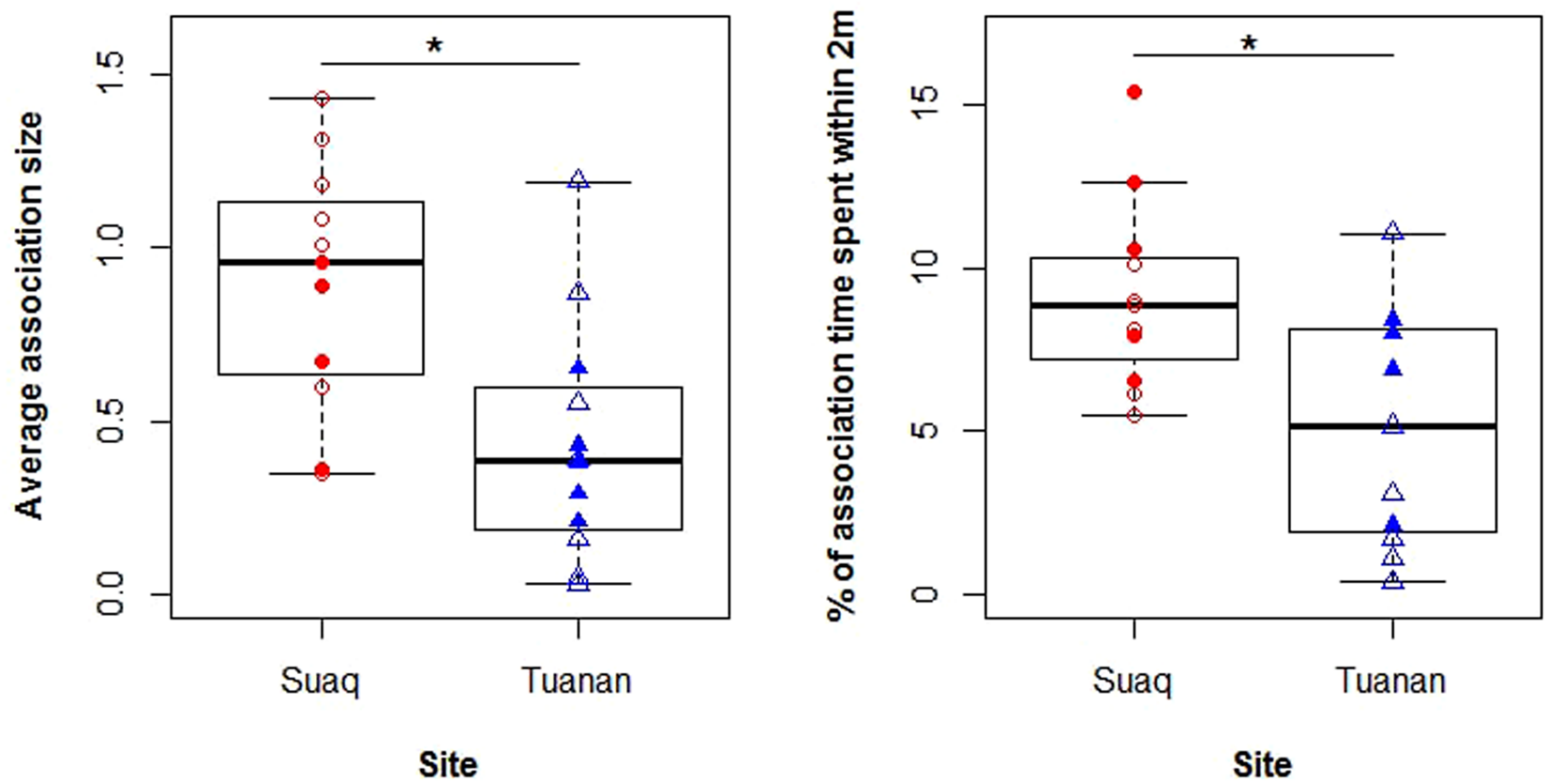
Average number of associates (excluding the mother and semi-dependent siblings) for dependent immatures at Suaq and Tuanan (**a**); and percent of association time spent within two meters of adult association partners other than the mother (**b**). Open symbols refer to dependent immatures from 0–2.9 years and filled symbols to dependent immatures from 3–6 years.

Being in association with conspecifics is the key precondition for social learning: once an individual is in association with a conspecific, depending on its social interest it can chose to approach another individual or not. Varying association frequencies and durations as well as varying levels of tolerance during these associations thus provide individuals with varying opportunities for social learning. Dependent immatures at Suaq had about twice the average number of association partners compared to dependent immatures at Tuanan, a significat difference (likelihood ratio test: p = 0.003, lmer: estimate = 0.46, Table 2_1a, Fig. 1a). Furthermore, immatures at Suaq spent significantly more time in close proximity of their association partners than immatures at Tuanan (likelihood ratio test: p = 0.008, lmer: estimate = 3.93, Table 2_1b, Fig. 1b). We divided dependent immatures into two age classes: from 0–2.9 years and 3–6 years because association patterns seem to vary with the age of the immatures.

**Table 2:** Linear mixed efect models with response variables, fixed effects and random effects: estimates, standard errors, *P-values, 95% confidence intervals, number of levels for the categorical variables (N Estimate) and total sample size of the model (N Total).

Nr	Response	Effect	Effect type	Estimate	Std-Error	P-value	Confidence Intervals		N Estimates	N Total
							**2.50%**	**97.50%**		
1a	Average number of association partners	Intercept	Fixed	0.80	0.14	—	0.53	1.06	—	23
		Age Class	Fixed (control)	0.16	0.14	0.210	−0.10	0.43	2	
		Site (Suaq)	Fixed (predictor)	0.46	0.16	0.003	−0.71	−0.20		
		Individual	Random	—	—	—	—	—	17 (23)	
1b	% of association time spent within 2 m	Intercept	Fixed	11.01	1.15	—	8.79	13.23		23
		Age Class	Fixed (control)	−3.02	1.08	0.015	−4.87	−0.68	2	
		Site (Suaq)	Fixed (predictor)	3.93	1.41	0.008	−6.09	−1.97	2	
		Individual	Random	—	—	—	—	—	17 (23)	
2a	log(Peering events per follow hour)	Intercept	Fixed	0.52	0.22	—	0.10	0.94	—	30
		Age	Fixed (control)	−0.21	0.03	<0.001	−0.27	−0.15	—	
		Site (Suaq)	Fixed (predictor)	1.25	0.24	<0.001	−1.71	−0.79	2	
		Individual	Random	—	—				19	
2b	sqrt(Peering events at the mother per time with mother)	Intercept	Fixed	1.06	0.07	—	0.95	1.22	—	30
		Age	Fixed (control)	−0.06	0.01	<0.001	−0.08	−0.03	cont.	
		Site (Suaq)	Fixed (predictor)	0.39	0.09	<0.001	−0.57	−0.26	2	
		Individual	Random	—	—	—	—	—	19	
2c	log(Peering events at others per time with them)	Intercept	Fixed	0.07	0.06	—	−0.05	0.19	—	30
		Age	Fixed (control)	0.07	0.03	0.005	0.02	0.12	cont.	
		Age^2^	Fixed (control)	−0.01	0.00	0.006	−0.01	0.00	cont.	
		Site (Suaq)	Fixed (predictor)	0.15	0.06	0.007	−0.26	−0.04	2	
		Individual	Random	—	—	—	—	—	19	
3	log(Peering events per item and time the mother was eating a specific item)	Intercept	Fixed	−1.15	0.08	—	−1.29	−0.99	—	115
		Complexity	Fixed (predictor)	0.09	0.03	0.003	0.03	0.15	5	
		Log Freq. in mother’s diet	Fixed (predictor)	−0.47	0.07	<0.001	−0.60	−0.34	cont.	
		Site (Suaq)	Fixed (predictor)	0.46	0.09	<0.001	−0.63	−0.31	2	
		Individual	Random	—	—	—	—	—	21	
4a	log(Exploratory object manipulation event per hour)	Intercept	Fixed	1.38	0.33	—	0.75	2.01	—	29
		Age	Fixed (control)	−0.24	0.03	<0.001	−0.30	−0.18	cont.	
		Sex (male)	Fixed (predictor)	0.01	0.28	0.963	−0.51	0.53	2	
		Site (Suaq)	Fixed (predictor)	0.62	0.22	0.005	−1.03	−0.20	2	
		Individual	Random	—	—	—	—	—	21	
4b	sqrt(Object play events per hour)	Intercept	Fixed	1.50	0.13	—	1.25	1.75	—	29
		Age	Fixed (control)	−0.12	0.01	<0.001	−0.14	−0.10	cont.	
		Sex (male)	Fixed (predictor)	0.11	0.11	0.275	−0.14	−0.10	2	
		Site (Suaq)	Fixed (predictor)	0.18	0.09	0.053	−0.35	−0.02	2	
		Individual	Random	—	—		—	—	21	
5	log(Exploratory object manipulation event per hour)	Intercept	Fixed	−2.36	0.20	—	−2.74	−1.97	—	24
		Age class (Indep. Imm.)	Fixed (control)	1.13	0.23	<0.001	0.68	1.57	2	
		Site (Suaq)	Fixed (predictor)	0.73	0.23	0.003	−1.17	−0.28	2	
		Individual	Random	—	—	—	—	—	21	
6	Exploratory object manipulation event per hour	Intercept	Fixed	0.11	0.04	—	0.04	0.18	—	46
		Age class (Indep. Imm.)	Fixed (control)	0.08	0.04	0.034	0.01	0.16	2	
		Site (Suaq)	Fixed (predictor)	0.09	0.04	0.022	−0.16	−0.01	2	
		Context (Social)	Fixed (predictor)	0.08	0.04	0.023	0.01	0.16	2	
		Individual	Random	—	—		—	——	21	

^*^P-values were calculated via the likelihood ratio test by excluding the specific factor and comparing the reduced model to the full model.

1: Average association time with individuals other than the mother or the semi- dependent sibling (a) and percent of association time spent within wo meters of adult association partners other than the mother (b) as response variable. The factor “Age class” refers to the two classes of dependent immatures: 0–2.9 y and 3–6 y.

2 c: Peering events at individuals other than the mother per hour spent in association with at least one of those individuals as response variable.

3: Peering rate as response variable (number of peering events per item and time the mother was eating a specific food item), number of pre-ingestion processing steps of the food item (“Complexity”), frequency of the food item in the mothers diet (log transformed; “Frequency”) as effects. The N model here represents the total number of individual - age - food item combinations.

5: The effect “Age class” refers to independent immatures versus mothers.

6: The effect “Age class” refers to independent immatures versus mothers and “Context” to alone (solitary) versus social (with at least one association partner other than the own dependent or semi- dependent offspring.

### Data Availability statement

All data used for the analyses of this article can be found in the supplementary material (Tables S1a & b).

### Ethical statement

As a strictly observational study on wild animals, there was no interaction with our study animals in any way. The research protocols were approved by the Ministry of research and technology (RISTEK; Research Permit No.: 152/SIP/FRP/SM/V/2012) and complied with the legal requirements of Indonesia.

## Results

### Prediction 1: Individuals at the more sociable population will make use of their increased opportunities for social learning and thus show higher rates of peering

**Figure 2:**
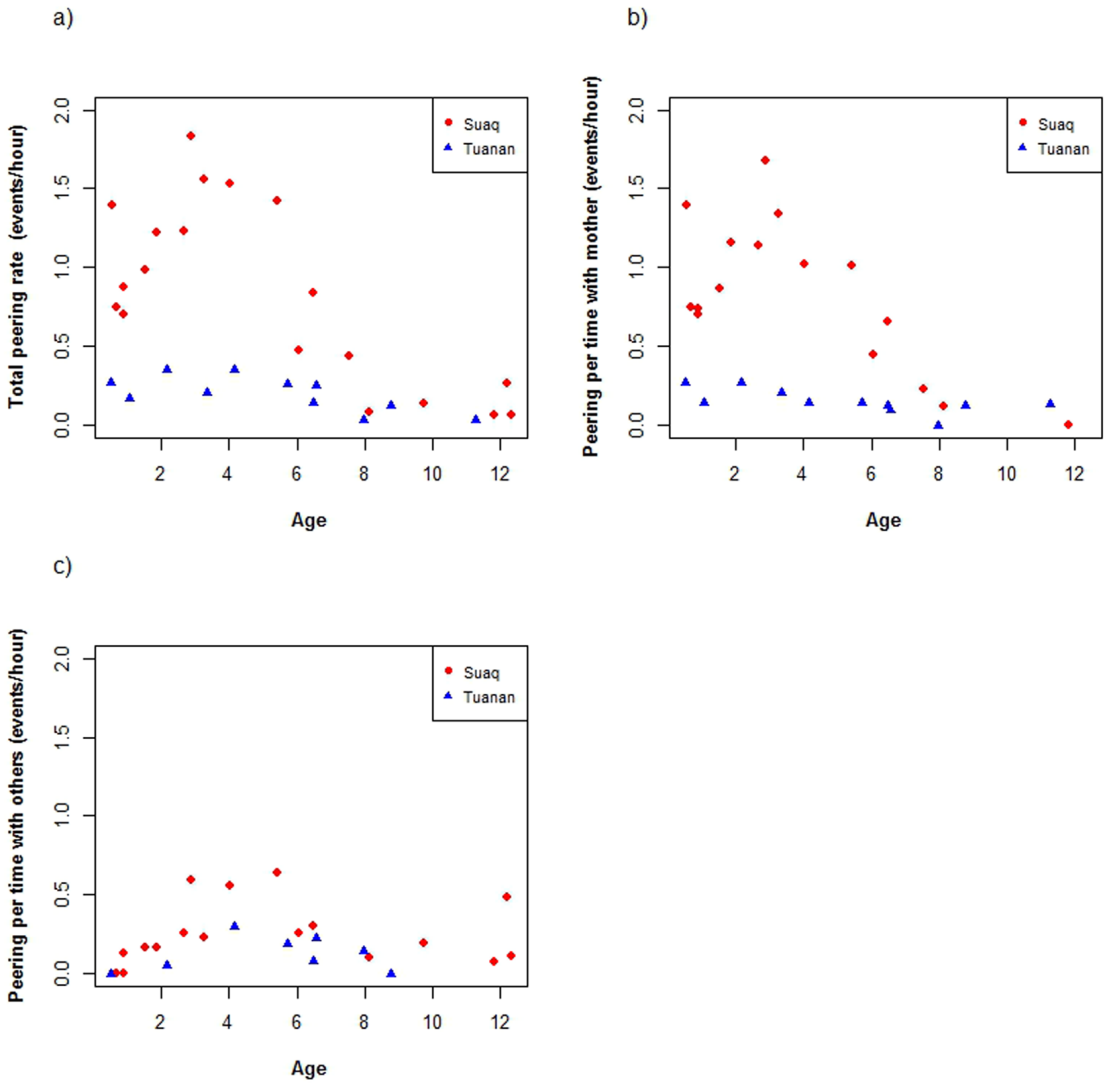
Average total peering rates (peering events per hour) versus age in years (**a**). Peering at the mother, corrected for time spent with her (peering events at the mother per hour spent with her) versus age (**b**). Peering at the other individuals, corrected for the time spent with them (peering events at other individuals than the mother per hour spent with them) versus age (**c**).

To investigate the use of increased opportunities for social learning, we first looked at the ontogeny of peering behaviour. We found that hourly peering rates (irrespective of context) increased until the age of 3–4 years and then decreased (Fig. 2a). Immatures at Suaq peered significantly more often per hour than immatures at Tuanan (likelihood ratio test: p < 0.001, lmer: estimate = 1.25, Table 2_2a, Fig. 2a). To correct for varying opportunities to peer, we also examined peering rates per time spent in association with different classes of individuals. Suaq immatures peered significantly more at their mother per hour spent in association with her than Tuanan individuals (likelihood ratio test: p < 0.001, lmer: estimate = 0.39, Table 2_2b, Fig. 2b). For peering at association partners other than the mother we found that peering rates per hour spent in association with those individuals began increasing from the age of 1.5–2 years, peaked around weaning and then decreased again. Suaq immatures peered significantly more frequently at association partners other than the mother per hour they spent with them than did Tuanan immatures (likelihood ratio test: p = 0.007, lmer: estimate = 0.15, Table 2_2c, Fig. 2c).

**Figure 3:**
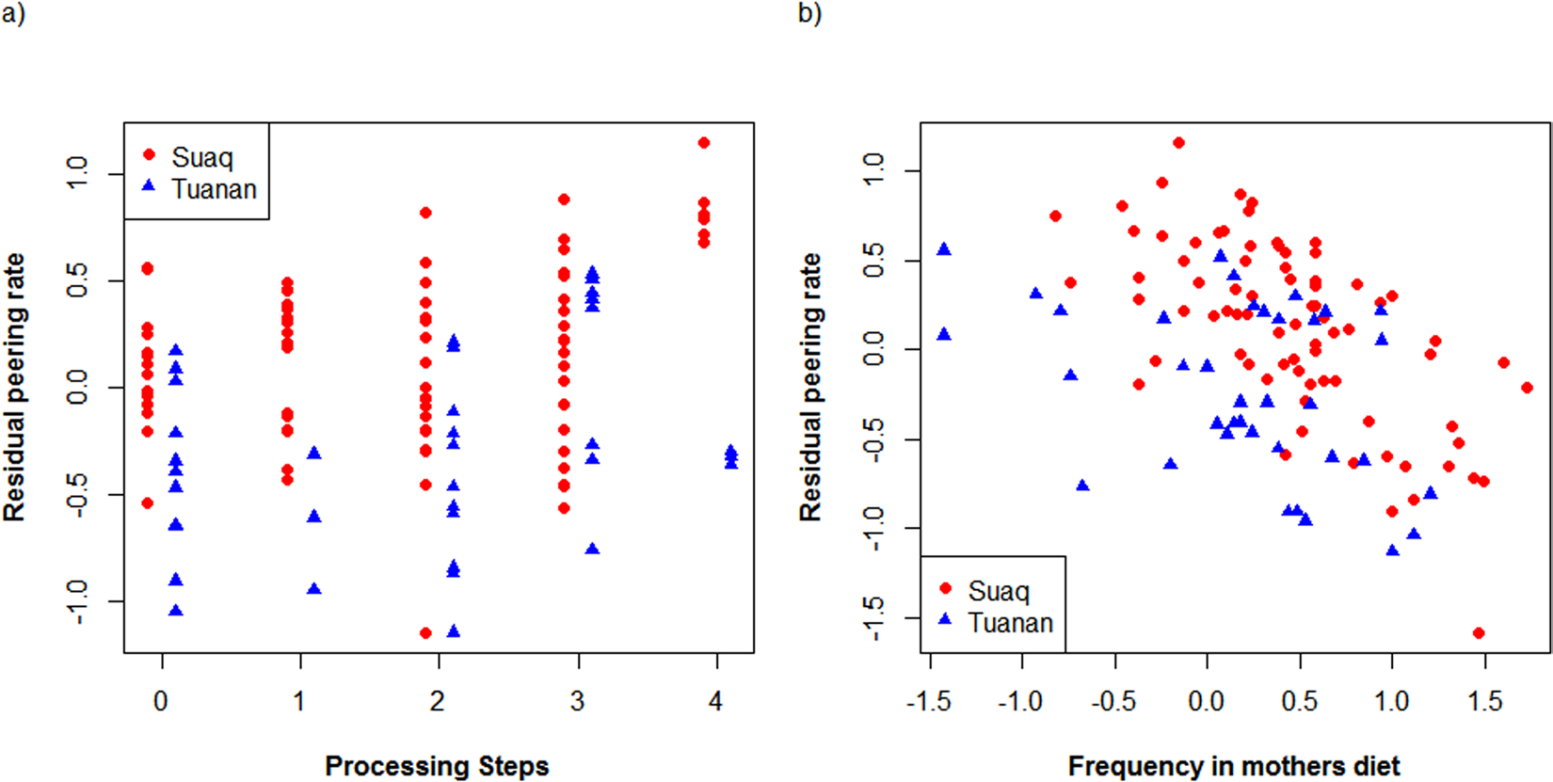
Population differences in immatures’ peering rates in relation to food complexity and frequency: Residual peering rates (corrected for frequency) as a function of complexity (**a**) and residual peering rates (corrected for complexity) as a function of log transformed frequency in the mother’s diet (**b**) for dependent immatures peering at their mothers in the feeding context at Suaq and Tuanan. Residuals were used for illustrative purposes only (see Table 2 for statistics).

Second, we examined differences in peering rates of the dependent immatures directed at their mothers in the feeding context. This measure automatically corrects for opportunities to peer because the mother is always available at both sites. Previously, we had found that peering rates were correlated with food item complexity (number of steps needed to process the food item, “complexity”) and the frequency of the food item in the mother’s diet (in percentage of total diet, “frequency”) (for definitions see^27^). To correct for the fact that food items are fed on for different amounts of time and the offspring therefore had varying opportunities to peer, peering rates were calculated as the number of peering events recorded for a given food item per unit time the mother was feeding on this item. To reduce the risk of outliers diluting the results, we only included food items that were fed on by the mother for a total of at least 20 minutes during an observation period. We included data of 6 infants at each site with a highly comparable age range (at both sites ranging from 0.5–6 y, with an average age of 2.9 and 3.0 y respectively). To reach evenly distributed residuals, the predictor variable “frequency” and the response variable (peering rate) were log transformed. We found, as expected^27^, that first, peering rates increased with increasing complexity of the food item (likelihood ratio test: p = 0.003, lmer: estimate = 0.09, Table 2_3, Fig. 3). Second, as predicted we found that dependent immatures peer less frequently with increasing frequency of the food item (likelihood ratio test: p < 0.001, lmer: estimate = −0.47,Table 2_3, Fig. 3). Third, even when correcting for frequency and rarity, Suaq infants showed significantly higher peering rates than Tuanan infants (likelihood ratio test: p < 0.001, lmer: estimate = 0.46, Table 2_3, Fig. 3).

### Prediction 2: Individuals at the more sociable population will show higher rates of exploratory object manipulation behaviour but not necessarily higher rates of non-exploratory object manipulation behaviour (object play). The difference in exploration should be most pronounced in immatures but also be retained in adults

**Figure 4:**
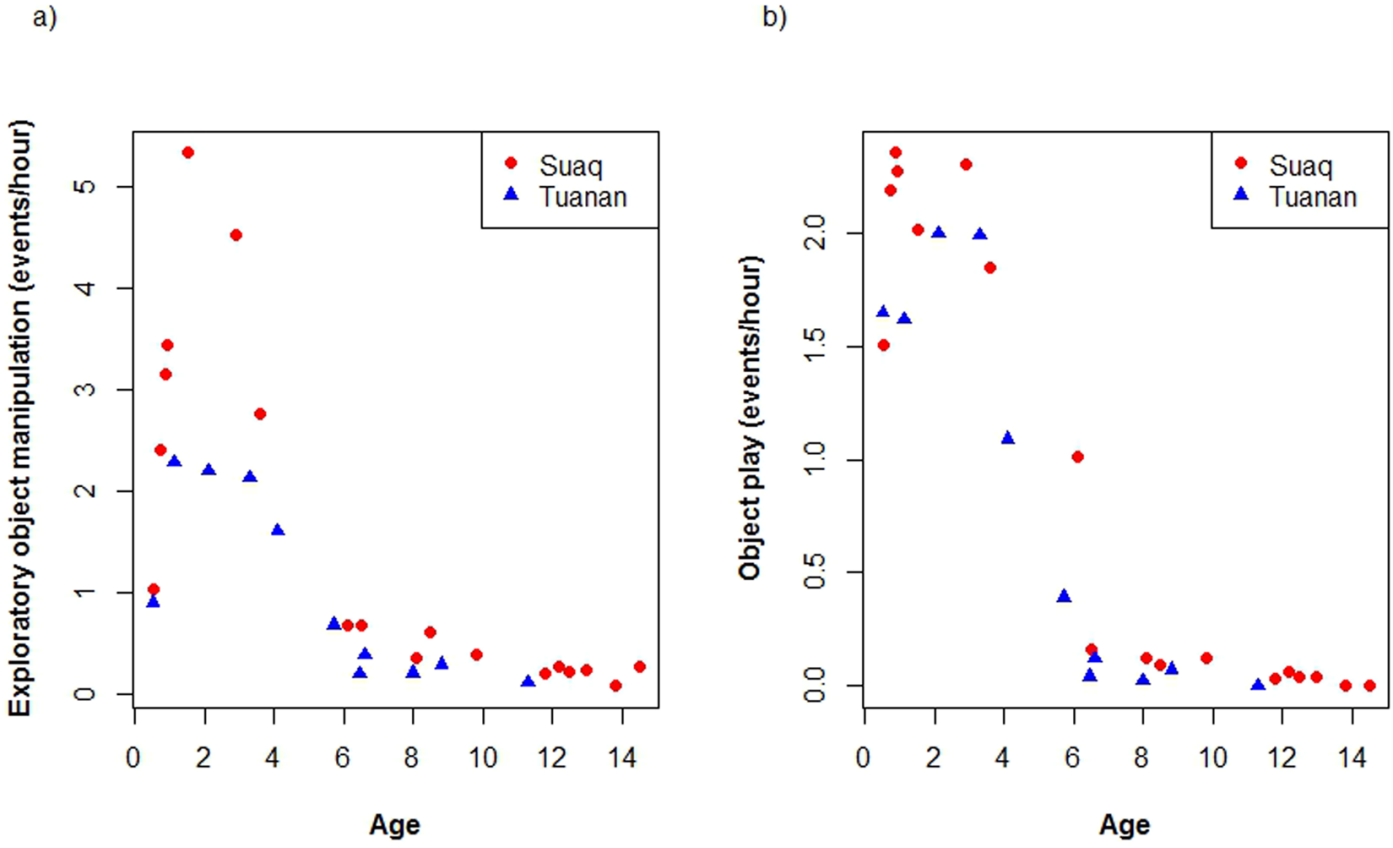
Average hourly rates of exploratory object manipulation (**a**) and object play (**b**) versus age for the immatures of Tuanan and Suaq.

Regarding population differences in exploratory tendency in immatures, we found that at both sites rates of exploratory object manipulation first increased with age, peaked around the age of 2–3 years and then decreased. By the age of weaning, the rates of these behaviours had dropped to around 0.5 events per hour. Immatures at Suaq showed significantly higher rates of exploration than immatures at Tuanan (likelihood ratio test: p = 0.005, lmer: estimate = 0.62, Table 2_4a, Fig. 4a). To rule out the possibility that this population difference could be caused by an overall higher level of activity of the Suaq immatures, we also looked at rates of object play behaviour. Although the same age trajectory was found as for exploratory object manipulation behaviour, there was only a trend for a difference in rates of object play manipulation between the populations (likelihood ratio test: p = 0.053, lmer: estimate = −0.18, Table 2_4b, Fig. 4b).

**Figure 5:**
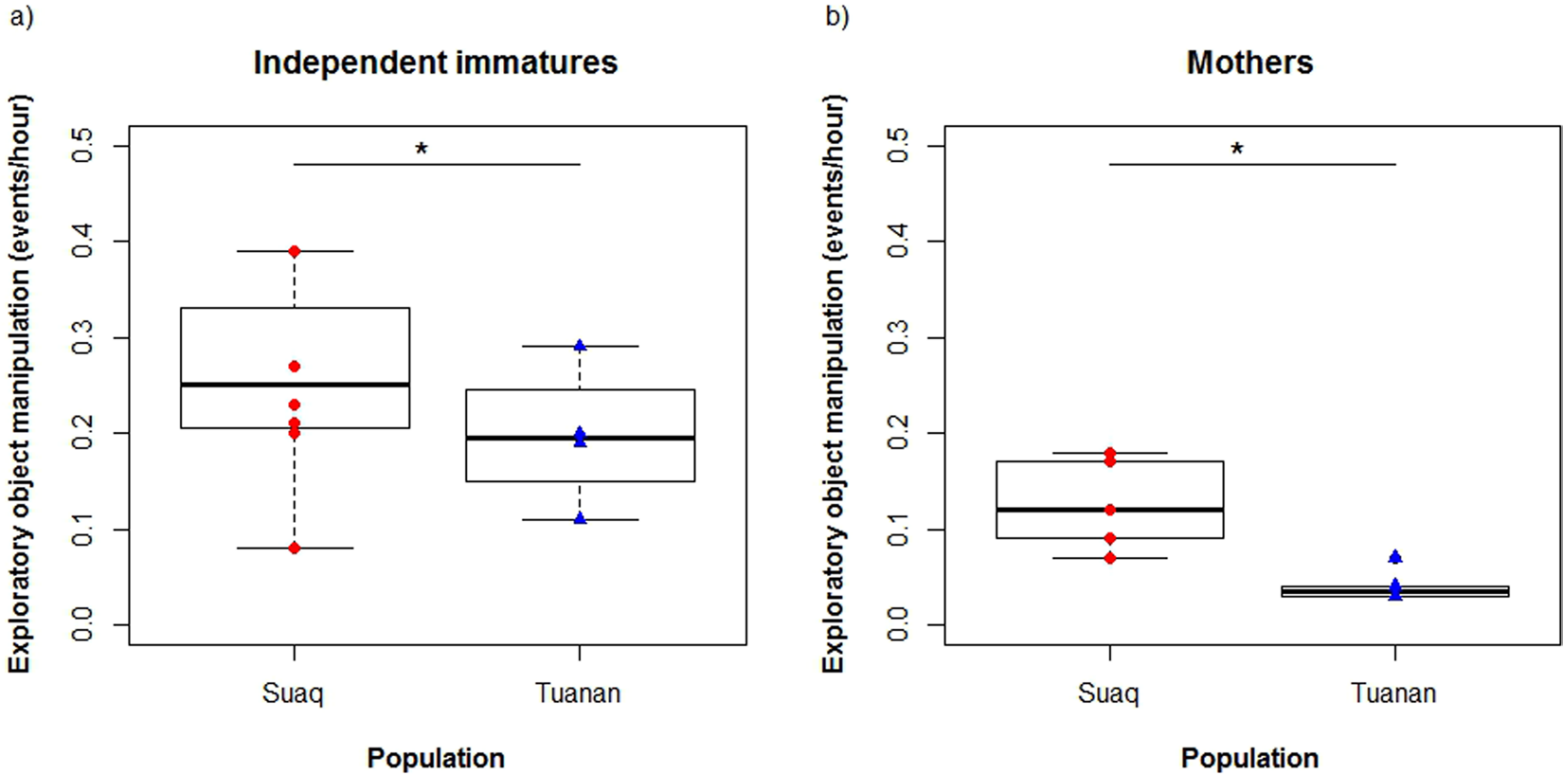
Rates of exploratory object manipulation (events per observation hour) for independent immatures (**a**) and adults (**b**) at Suaq versus Tuanan.

To assess whether these early differences in exploratory tendency have lasting effects, we also compared rates of exploratory object manipulation in independent immatures and adults. We found that rates of exploratory object manipulation were significantly higher in Suaq than in Tuanan (likelihood ratio test: p_ = _0.003, lmer: estimate = 0.73, Table 2_5, Fig. 5a,b).

### Prediction 3: If associations have direct effects on exploration, individuals should increase their exploration rates when in association. However, under the assumption of developmental effects on exploratory behavior, the population difference in exploration should remain in the solitary context

**Figure 6:**
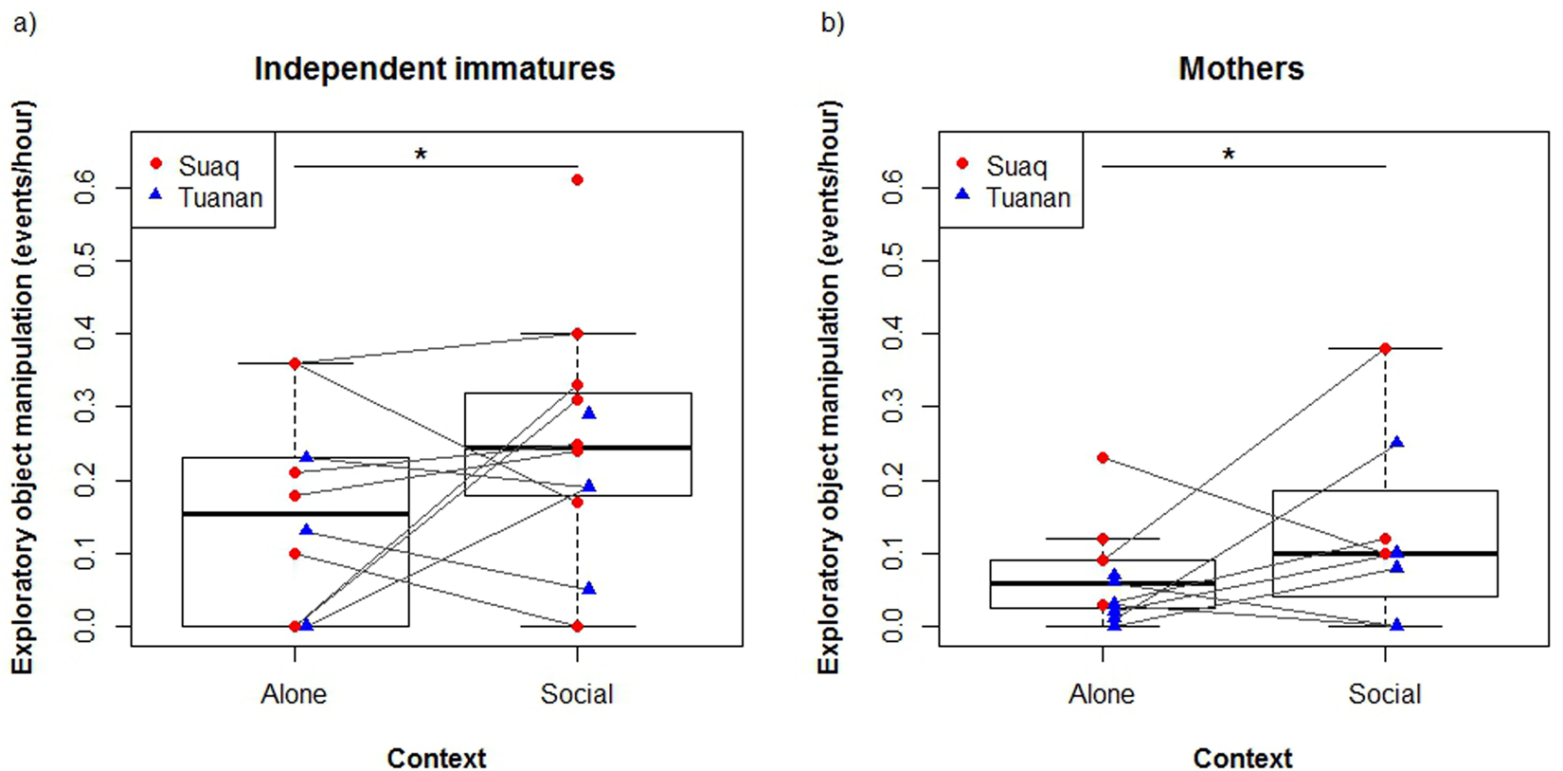
Rates of exploratory object manipulation (events per hour) in the solitary context (when being alone or with dependent or semi-dependent offspring only, “Alone”) and in the social context (when being with at least one association partner that is not the own dependent or semi dependent offspring, “Social”) for independent immatures at Suaq and Tuanan (**a**), as well as for adults at Suaq and Tuanan (**b**).

We compared rates of exploratory object manipulation of adults and independent immatures in both populations when in association with individuals (social context) other than their own dependent and semi-dependent immatures to when being solitary (solitary context). We found that at both populations, individuals showed significantly higher exploration rates when being in association with others compared to when being on their own (likelihood ratio test: p = 0.023, lmer: estimate = 0.08, Table 2_6, Fig. 6).

We subsequently tested whether the higher rates of exploratory object manipulation at Suaq than at Tuanan (see above) merely reflected this immediate result of increased assoctiation frequency. To do so, we looked at rates of exploration in the solitary context, where direct effects of associations can be excluded. We compared rates of exploratory object manipulation of mothers of both populations when solitary and found that mothers at Suaq still showed significantly higher exploration rates than Tuanan mothers (Lm: N = 11, B_site_ = −0.08, Stde_site_ = 0.03, P_site_ = 0.034, R^2^ = 0.41), thus confirming a consistent population difference in exploration tendency.

### Prediction 4: Ultimately, higher exploratory tendency will be correlated with more complex and larger repertoires of innovations at the more sociable population

**Figure 7:**
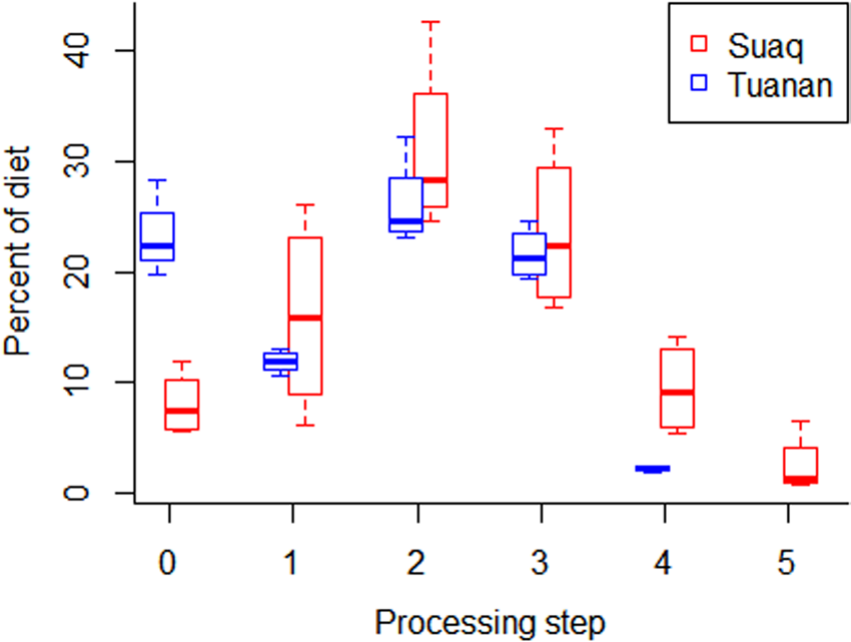
Percentage of the different processing steps in the diets of 4 adult females at Suaq and Tuanan each.

To test whether increased exploratory tendencies will be correlated with a higher likelihood of innovation and thus more complex and larger repertoires at the level of the population, we first compared the distributions of the different processing steps at the two sites (Fig. 7). This comparison was based on the diets of the 4 adult females with the most data available at each site (Tuanan: 4098–5161 hours, mean = 4644 hours, Suaq: 320–1168 hours, mean = 679). We found that the distributions of the different processing steps differed significantly between the two sites (Kolmogorov-Smirnov: D = 0.184, P < 0.001). We found that Suaq individuals had a higher share of processing steps 4 and 5 as well as a higher average dietary complexity than Tuanan individuals. Accordingly, Tuanan individuals had a higher share of processing step 0 than Suaq individuals (Fig. 7).

Second, as a measure of repertoire size we compared the number of insect feeding techniques at the two sites. Insects are ubiquitously available and eaten at both sites and are thus the ideal comparison. We found that at Suaq individuals use 17 different techniques to feed on insects whereas at Tuanan only 8 of these techniques (largely a subset) occur. One additional technique was only seen in Tuanan but not in Suaq, (Table S2) where it could not be performed because of ecological constraints. All other techniques are unlikely to be influenced by ecological factors since both the insect types and the relevant substrates occur at both sites.

## Discussion

To investigate the connection between sociability and current motivation to explore, we compared two populations of orang-utans on how differences in opportunities for social learning affected the exploratory tendency of adults and immatures. In line with our predictions, we first found that immatures in the more sociable population (Suaq, Sumatra) did indeed make use of their increased opportunities for social learning by showing significantly higher rates of peering than same-aged individuals at the less sociable population at Tuanan (Borneo). Second, both immatures and adults at Suaq showed significantly higher rates of exploratory behaviour compared to Tuanan individuals. And finally, we found evidence that the diet in the more sociable population at Suaq is both more diverse and more complex, in line with the notion that increased exploration will lead to more innovations.

The higher peering rates at Suaq suggest a higher motivation to learn from conspecifics. This could be caused by either of two non-exclusive mechanisms: On the one hand, the exposure to a more diverse and complex skill set of one’s conspecifics may directly trigger higher peering rates. On the other hand, larger and more complex skill sets may have selected for a genetically anchored increased level of social interest: in a population where there is more to learn, increased attention to conspecifics will pay off because it improves the chances of learning a novel technique. To disentangle these two possibilities, we compared peering rates between the two populations while controlling for the complexity and frequency of the food items, which had previously been shown to affect peering rates^27^. Our results indicate that even when controlling for these two factors, immatures at Suaq show significantly higher baseline peering rates, suggesting that the greater social attentiveness ofthe Suaq individuals reflects an intrinsic predisposition or lasting (organisational) effects of increased exposure to large skill repertoires.

On top of being more attentive to their conspecifics behaviour, the immatures at Suaq were also found to be more exploratory, as predicted. Among immatures, the population difference in exploratory behaviour was much bigger than the insignificant difference observed in object play, implying that the difference in exploratory behaviour was not due to a general increase in activity in the more sociable population. Even though exploration rates dropped to very low levels by the end of juvenility, the population difference in exploration rates was also found among the adult individuals, suggesting very fine-grained effects.

Various factors may contribute to causing the variation in exploration tendency. The finding that rates of exploratory behaviour were higher when individuals were in association with others than when alone implies a direct effect of being in association on exploration. Thus, it is likely that the difference in exploratory tendency found between the sites are caused by the difference in sociability between the sites. Experimental studies of effects of the presence of conspecifics on exploration in several mammal and bird species have produced mixed results: some have found negative effects of associations, ascribing them to socially induced neophobia or the possibility that social interactions simply lead to a decrease of the amount of time that can be devoted to other activities^49–52^. Others have found positive effects of conspecifics’ presence on exploration, and suggested reduced stress, a reduced need for vigilance, or social facilitation effects as underlying mechanisms^53,54^.

Our finding that orang-utan mothers’ exploration rates only increased when in association with individuals other than their own dependent or semi-dependent offspring could be explained by a selective social facilitation effect by individuals that are perceived as more knowledgeable than oneself. However, the finding is also in line with a vigilance effect: since young immature individuals are unlikely to contribute to shared vigilance, being in association with dependent offspring does not lead to reduced vigilance demands on the mother. At present, we cannot differentiate between these two non-exclusive explanations. We conclude that association with adult individuals increases exploration in orang-utans due to a socially facilitated reduction in neophobia and/ or a reduced cognitive load and that some of the between-population difference is due to this phenomenon.

Our results also provided evidence for indirect positive effects of increased sociability on independent exploratory tendency. The differences in exploration rates between the populations persisted after controlling for differences in association time: even when solitary, independent immatures and mothers at Suaq showed higher rates of exploration compared with Tuanan individuals. Two non-exclusive causes could underlie this difference. First, they may reflect intrinsic differences in exploratory tendency between the populations. A recent study showed that Sumatran orang-utans in zoos, despite very similar housing conditions, showed differences in exploration style and accordingly a higher problem-solving success than Bornean orang-utans, suggesting intrinsic species differences^55^. Second, the increased exploratory tendency could be a lasting (organisational) effect of exposure to more and more complex skills in early life, which may make infants keener to learn and thus explore. In line with this scenario, after their reintroduction into the wild, rehabilitated great apes produce various innovations that have never been seen in the wild^56^, suggesting that increased social inputs during their time with humans have lasting consequences for exploration (cf. Damerius *et al*. in prep.). All in all, these developmental effects of being exposed to a different numbers of role models with varying skill repertoires are comparable to developmental niche construction in humans, where the construction of a learning environment by culturally knowledgeable others affect infant development^57^. We expect that both intrinsic and developmental factors contribute to the observed population difference in both exploratory tendency and rate of social learning. Data on more wild populations are needed to confirm an intrinsic species difference in exploratory tendency and its link with sociability. Also, examination of the within-population variation of peering rates and exploratory tendency will allow us to further evaluate the strength of the immediate effect of exposure versus intrinsic predispositions.

The increased exploratory tendency at Suaq, regardless of its cause, implies higher innovation rates there, which should ultimately lead to a larger skill pool in the Suaq population, provided that these innovations are successfully transmitted. Also, increased rates of social learning at Suaq (as suggested by the increased peering rates) imply increased transmission and retention rates and thus a faster accumulation of skills and the presence of more complex skills in this population^21^. Accordingly, we found that diet repertoires at Suaq are more complex than the ones at Tuanan, that the most complex skills such as tool use are only found at Suaq^60^, and that the repertoire of learned insects-feeding skills at Suaq is about twice as large as the repertoire at Tuanan. Ecological differences between two sites may of course contribute to differences in behavioural repertoires and thus, ecological explanations cannot be ruled out completely. However, in the specific case of insect feeding, it is unlikely that the observed population difference is solely caused by ecological differences since both sites have the same or very similar insects. Also, there is no difference in the opportunities for the use of the most complex insect feeding technique, namely tool use: tree hole abundance and occupancy are broadly similar at the two sites (Schuppli unpublished data).

## Conclusions

In conclusion, we found evidence for effects of sociability and opportunities for social learning in orang-utans on two levels. First, associations lead to a direct increase of exploration rates. Second, between populations, increased sociability levels during infancy seem to positively affect subsequent independent exploratory tendencies, and this effect lasts into adulthood. We found that ultimately, this is connected to higher innovation frequencies and thus for the larger and more complex population skill repertoires observed among the individuals of the more sociable population. To confirm these between-population effects of increased sociability on exploratory tendency, behavioural data on more wild populations will be needed. However, our findings provide support from natural great ape populations for the idea developed for humans that growing up with increased opportunities for social learning from a larger number of role models triggers a cascade, from increased levels of social learning, through increased levels of socially triggered practice to an increase in exploratory tendency and ultimately a larger skill pool.

## Electronic supplementary material

Supplementary data
