# Supplementary material for: The effects of sociability on exploratory tendency and innovation repertoires in wild Sumatran and Bornean orangutans
Source: Sci Rep. 2017 Nov 13;7:15464. doi: 10.1038/s41598-017-15640-x (PMC5684228; doi:10.1038/s41598-017-15640-x)
Supplement: Supplementary file 1 — Supplementary data [file 41598_2017_15640_MOESM1_ESM.doc]

**The effects of sociability on exploratory tendency and innovation repertoires in wild Sumatran and Bornean orangutans**

Caroline Schuppli^1*^, Sofia Forss^1^, Ellen Meulman^1^, Suci Utami Atmoko^2^, Maria van Noordwijk^1^ and Carel van Schaik^1^

*Corresponding author: caroline.schuppli@aim.uzh.ch

1: Department of Anthropology, University of Zürich, Winterthurerstrasse 190, 8057 Zürich, Switzerland

2: Fakultas Biologi, Universitas Nasional, Jl. Sawo Manila, RT.14/RW.3, Ps. Minggu, DKI Jakarta,, Indonesia

**Supplementary Material**

**Table S1a. Data used for the analyses of prediction 1 (figure 2a-c, table S2_2a-c): site, focal animal, sex, age, age class, total follow time, total peering rate (number of peering events per follow hour), total time spent in association with the mother, peering rate at the mother (number of peering events directed at the mother per hour spent with her), total time spent in association with other individuals than the mother, peering rate at other individuals (number of peering events directed at other individuals per hour spent with them). Associations were defined as being within 50 meters of another individual.**

| **Site** | **Individual** | **Sex** | **Age (y)** | **Age class** | **Total follow time (h)** | **Peering rate (events/ h)** | **Time with mother (h)** | **Peering at mother rate (events/ h)** | **Time with others (h)** | **Peering at others rate (events/ h)** |
| --- | --- | --- | --- | --- | --- | --- | --- | --- | --- | --- |
| Suaq | Lois | M | 0.53 | infant | 110.8 | 1.40 | 110.8 | 1.40 | 4.7 | NA |
| Suaq | Rendang | M | 0.69 | infant | 39.9 | 0.75 | 39.9 | 0.75 | >0 | 0.00 |
| Suaq | Simba | M | 0.89 | infant | 59.3 | 0.71 | 59.3 | 0.71 | >0 | 0.00 |
| Suaq | Frankie | M | 0.91 | infant | 110.5 | 0.88 | 110.5 | 0.74 | 110.5 | 0.14 |
| Suaq | Cinnamon | F | 1.51 | infant | 87.2 | 0.99 | 87.2 | 0.87 | 60.6 | 0.17 |
| Suaq | Ronaldo | M | 1.86 | infant | 73.7 | 1.22 | 73.7 | 1.15 | 29.8 | 0.17 |
| Suaq | Fredy | M | 2.68 | infant | 77.3 | 1.23 | 77.3 | 1.14 | 27.5 | 0.25 |
| Suaq | Lois | M | 2.88 | infant | 83.4 | 1.83 | 83.4 | 1.68 | 22.0 | 0.59 |
| Suaq | Fredy | M | 3.26 | infant | 100.0 | 1.56 | 100.0 | 1.34 | 94.0 | 0.23 |
| Suaq | Fredy | M | 4.04 | infant | 58.8 | 1.53 | 58.8 | 1.02 | 54.0 | 0.56 |
| Suaq | Chindy | F | 5.41 | infant | 60.3 | 1.43 | 60.3 | 1.01 | 38.9 | 0.64 |
| Suaq | Fredy | M | 6.06 | infant | 112.6 | 0.48 | 112.6 | 0.45 | 11.7 | 0.26 |
| Suaq | Lilly | F | 6.49 | infant | 99.1 | 0.84 | 99.1 | 0.66 | 58.6 | 0.31 |
| Suaq | Lilly | F | 7.55 | infant | 29.8 | 0.44 | 29.8 | 0.24 | 0.0 | NA |
| Suaq | Chindy | F | 8.14 | indep. im. | 48.1 | 0.08 | 25.2 | 0.12 | 9.8 | 0.10 |
| Suaq | Lilly | F | 9.76 | indep. im. | 77.9 | 0.14 | 0.0 | NA | 56.2 | 0.20 |
| Suaq | Ellie | F | 11.84 | indep. im. | 63.6 | 0.06 | 1.3 | 0.00 | 54.5 | 0.07 |
| Suaq | Shera | F | 13.00 | indep. im. | 100.6 | 0.27 | 0.0 | NA | 55.8 | 0.48 |
| Suaq | Lilly | F | 12.34 | indep. im. | 59.6 | 0.07 | 0.0 | NA | 34.1 | 0.12 |
| Tuanan | Kahiyu | F | 0.50 | infant | 114.8 | 0.27 | 114.8 | 0.27 | >0 | 0.00 |
| Tuanan | Joya | M | 1.06 | infant | 78.2 | 0.17 | 78.2 | 0.14 | 5.1 | NA |
| Tuanan | Danum | M | 2.14 | infant | 122.0 | 0.35 | 122.0 | 0.27 | 41.4 | 0.05 |
| Tuanan | Joya | M | 3.34 | infant | 78.0 | 0.21 | 78.0 | 0.21 | 0.0 | NA |
| Tuanan | Mawas | F | 4.13 | infant | 122.4 | 0.35 | 122.4 | 0.15 | 43.2 | 0.30 |
| Tuanan | Kino | M | 5.71 | infant | 109.2 | 0.27 | 109.2 | 0.15 | 69.9 | 0.19 |
| Tuanan | Jip | M | 6.59 | infant | 140.0 | 0.25 | 140.0 | 0.09 | 97.9 | 0.22 |
| Tuanan | Deri | M | 7.99 | indep. im. | 53.7 | 0.04 | 41.1 | 0.00 | 32.8 | 0.06 |
| Tuanan | Milo | F | 11.29 | indep. im. | 97.7 | 0.03 | 15.3 | 0.13 | 0.0 | NA |

**Table S1b.** Data used for the analyses of prediction 2 & 3 (figures 4 a&b, 5a&b, 6 , tables S2_ 4 a&b, S2_5a&b, S2_6): site, focal animal, sex, age, age class, total follow time, total time spent in association, total time spent solitary (for mothers and independent juveniles only, for mothers excluding their dependent and semi dependent offspring as association partners), exploration rate (number of exploration events per follow hour), social exploration rate (number of exploration events during associations per hour spent in association), solitary exploration rate (number of exploration events during solitary time per hour spent solitarily), object play rate (number of object play event per follow hour. Associations were defined as being within 50 meters of another individual. The differentiation in solitary and social time is not applicable to infants because they spend 100% of their time in association with their mothers.

| **Site** | **Individual** | **Sex** | **Age (y)** | **Age class** | **Total follow time (h)** | **Total time social (h)** | **Total time solitary (h)** | **Exploration rate (events/h)** | **Social exploration rate (events/h)** | **Solitary exploration rate (events/h)** | **Object play rate (events/ h)** |
| --- | --- | --- | --- | --- | --- | --- | --- | --- | --- | --- | --- |
| Suaq | Lois | M | 0.53 | infant | 77.4 |  |  | 1.03 |  |  | 1.51 |
| Suaq | Rendang | M | 0.68 | infant | 52.7 |  |  | 2.41 |  |  | 2.19 |
| Suaq | Frankie | M | 0.90 | infant | 183.9 |  |  | 3.43 |  |  | 2.28 |
| Suaq | Simba | M | 0.88 | infant | 117.5 |  |  | 3.15 |  |  | 2.36 |
| Suaq | Cinnamon | F | 1.51 | infant | 102.6 |  |  | 5.33 |  |  | 2.02 |
| Suaq | Lois | M | 2.88 | infant | 103.3 |  |  | 4.51 |  |  | 2.31 |
| Suaq | Fredy | M | 3.60 | infant | 75.4 |  |  | 2.76 |  |  | 1.85 |
| Suaq | Fredy | M | 6.05 | infant | 74.5 |  |  | 0.67 |  |  | 1.01 |
| Suaq | Lilly | F | 6.48 | infant | 69.8 |  |  | 0.67 |  |  | 0.16 |
| Suaq | Chindy | F | 8.13 | indep. im. | 75.2 | 38.9 | 36.3 | 0.36 | 0.43 | 0.26 | 0.12 |
| Suaq | Diddy | M | 8.50 | indep. im. | 11.5 | 11.5 | 0.0 | 0.61 | 0.61 | NA | 0.09 |
| Suaq | Lilly | F | 9.76 | indep. im. | 72.3 | 50.3 | 22.0 | 0.39 | 0.40 | 0.36 | 0.12 |
| Suaq | Ellie | F | 11.83 | indep. im. | 69.4 | 24.6 | 45.0 | 0.20 | 0.24 | 0.18 | 0.03 |
| Suaq | Lilly | F | 12.20 | indep. im. | 51.6 | 37.7 | 13.9 | 0.27 | 0.17 | 0.36 | 0.06 |
| Suaq | Lilly | F | 12.50 | indep. im. | 23.4 | 15.0 | 8.4 | 0.21 | 0.33 | 0.00 | 0.04 |
| Suaq | Shera | F | 13.00 | indep. im. | 69.4 | 40.7 | 28.7 | 0.23 | 0.25 | 0.21 | 0.04 |
| Suaq | Tina | F | 13.80 | indep. im. | 24.1 | 3.2 | 21.0 | 0.08 | 0.00 | 0.10 | 0.00 |
| Suaq | Ellie | F | 14.50 | indep. im. | 7.4 | 6.5 | 1.0 | 0.27 | 0.31 | 0.00 | 0.00 |
| Suaq | Cissy | F | adult | adult | 65.7 | 30.7 | 35.1 | 0.17 | 0.10 | 0.23 | 0.00 |
| Suaq | Friska | F | adult | adult | 69.4 | 0.0 | 69.4 | 0.09 | NA | 0.09 | 0.00 |
| Suaq | Lisa | F | adult | adult | 96.3 | 28.8 | 67.6 | 0.18 | 0.38 | 0.09 | 0.00 |
| Suaq | Raffi | F | adult | adult | 24.5 | 0.0 | 24.5 | 0.12 | NA | 0.12 | 0.00 |
| Suaq | Sarabi | F | adult | adult | 59.3 | 24.0 | 35.2 | 0.07 | 0.12 | 0.03 | 0.00 |
| Tuanan | Kahiyu | M | 0.50 | infant | 115.9 |  |  | 0.90 |  |  | 1.65 |
| Tuanan | Joya | M | 1.06 | infant | 71.2 |  |  | 2.28 |  |  | 1.62 |
| Tuanan | Danum | M | 2.14 | infant | 114.8 |  |  | 2.19 |  |  | 2.00 |
| Tuanan | Joya | M | 3.34 | infant | 77.6 |  |  | 2.12 |  |  | 1.99 |
| Tuanan | Mawas | F | 4.13 | infant | 122.4 |  |  | 1.60 |  |  | 1.09 |
| Tuanan | Kino | M | 5.70 | infant | 109.2 |  |  | 0.68 |  |  | 0.39 |
| Tuanan | Mawas | F | 6.46 | 1/2 indep. im. | 78.5 | 78.5 |  | 0.19 | 0.19 | 0.00 | 0.04 |
| Tuanan | Jip | M | 6.58 | infant | 110.9 |  |  | 0.38 |  |  | 0.12 |
| Tuanan | Deri | M | 8.00 | indep. im. | 53.7 | 32.0 | 21.7 | 0.20 | 0.19 | 0.23 | 0.02 |
| Tuanan | Jip | M | 8.80 | indep. im. | 74.6 | 74.6 | 0.0 | 0.29 | 0.29 | NA | 0.07 |
| Tuanan | Milo | F | 11.29 | indep. im. | 106.3 | 20.9 | 86.6 | 0.11 | 0.05 | 0.13 | 0.00 |
| Tuanan | Desy | F | adult | adult | 66.3 | 3.0 | 63.3 | 0.04 | 0.00 | 0.06 | 0.00 |
| Tuanan | Jinak | F | adult | adult | 77.6 | 9.8 | 67.7 | 0.03 | 0.10 | 0.02 | 0.00 |
| Tuanan | Juni | F | adult | adult | 74.6 | 4.0 | 70.6 | 0.03 | 0.25 | 0.01 | 0.00 |
| Tuanan | Kerry | F | adult | adult | 46.3 | 23.7 | 22.6 | 0.04 | 0.08 | 0.00 | 0.00 |
| Tuanan | Kondor | F | adult | adult | 55.4 | 0.0 | 55.4 | 0.07 | NA | 0.07 | 0.00 |
| Tuanan | Mindy | F | adult | adult | 78.5 | 7.4 | 71.0 | 0.03 | 0.00 | 0.03 | 0.00 |

**Table S2.** Different techniques to feed on invertebrates (ants, termites, bees and stingless bees): variants, descriptions and occurrence at the study sites. Yes= present at the site, No= never observed at the site, NoEcol.= Absent for ecological reasons, Yes? = unclear if present, indicated by anecdotal observations by observers other than the authors.

| **Technique** | **Variants** | **Description** | **Suaq** | **Tuanan** |
| --- | --- | --- | --- | --- |
| Bouqet feeding |  | Picking ants or termites from a fistful of dry or fresh leaves with lips. | Yes | Yes |
| Dead twig split lick |  | Splitting hollow twigs lengthwise using teeth and hands and then licking the ants out of them. | Yes | No |
| Dead twig sucking |  | Breaking hollow (dead) twigs using mouth and hands, then suck the ants out of the endings. | Yes | Yes? |
| Dripping out of nest | a) Hand b) Mouth | Dripping termites or ants out of their nest into the open hand (a) or directly into mouth (b). | Yes | Yes |
| Finger dipping |  | Catching termites or ants from leaves/ branches by touching the leaves with the fingertips of the stretched hand and letting them crawl onto the fingers. | Yes | Yes |
| Finger picking |  | Picking termites or ants off a surface (tree trunk or branches) using the thumb against the side of the index finger. | Yes | No |
| Fist fishing | a) Side of fist b) Front of fist | Wiping termites or ants with the fist off a surface (tree trunk or branches) using either the side of the fist (along pinkie finger, a) or front of the fist (along curled fingers, b). Then licking the insects off the hand/ wrist. | Yes | No |
| Fist smashing | a) Front of fist b) Bottom of fist  c) Back of wrist | Smashing bees or stingless bees against a surface with the front or the side of the fist or the back of the wrist. | Yes | No |
| Hollow fist leaf strip |  | Drawing leaves through the partly closed hand to obtain termites or ants that are on the foliage. | Yes | No |
| Licking |  | Licking termites or ants off a surface (tree trunk, branches) with protruded under lip. | Yes | Yes |
| Lip picking |  | Picking termites or ants off a surface (tree trunk, branches, leaves, old nest material) with lips (between upper and under lip). | Yes | Yes |
| Mouth foliage strip |  | Drawing leaves through the mouth to obtain termites or ants that are on the foliage. | Yes | No |
| Nest destruction |  | Rummaging through old orangutan nests for termites or ants, taking the nest apart while doing so. | Yes | Yes? |
| Smashing flying insects into fur |  | Catching bees or stingless bees by smashing them against own fur (mostly upper arm). | Yes | No |
| Snatching flying insects in the air | a) Full circle snatch b) Quick snatch | Catching bees or stingless bees in the air by snatching them with hand. | Yes | No |
| Soaking log in water |  | Soaking a piece of dead wood into the water of a stream or puddle to get the termites hidden inside to emerge to the surface of the wood. | No (Ecol.) | Yes |
| Sucking out of dead wood | a) Sucking b) Chewing | Eating termites or ants out of dead wood (log or dead tree trunk) by biting or breaking open dead wood, then sucking them out (a) or passing (chewing) pieces of dead wood through the mouth (b). | Yes | Yes |
| Tool use | Several different variants | Obtaining insects or their products by using tools: mainly inserting processed sticks into tree holes and insect nests. | Yes | No |
| **Total number of techniques** |  |  | **17** | **9** |
